# Correlation between field triage criteria and the injury severity score of trauma patients in a French inclusive regional trauma system

**DOI:** 10.1186/s13049-019-0652-0

**Published:** 2019-08-05

**Authors:** Arnaud Cassignol, Julien Marmin, Jean Cotte, Mickael Cardinale, Julien Bordes, Vanessa Pauly, François Kerbaul, Didier Demory, Eric Meaudre

**Affiliations:** 1 SMUR Department, Sainte-Musse Public Hospital, 83100 Toulon, cedex 9 France; 2 Prehospital Emergency Medical Services of Marine Fire Battalion, Marseille, France; 3 Anesthesia and Intensive Care Department, Sainte-Anne Military Hospital, 83041 Toulon, France; 4 Public Health and Medical Information Service, Conception Hospital, Aix-Marseille University, 13005 Marseille, France; 5 SMUR department, Timone Hospital, Aix-Marseille University, 13005 Marseille, France; 6 UMR MD 2, Aix-Marseille University, Marseille, France; 7 Clinical research unit, Sainte-Musse Public Hospital, 83100 Toulon, cedex 9 France

**Keywords:** Field triage, Vittel criteria, Algorithm, ISS, Mortality, Intensive care unit

## Abstract

**Background:**

In France, the pre-hospital field triage of trauma patients is currently based on the Vittel criteria algorithm. This algorithm was originally created in 2002 before the stratification of trauma centers and, at the national level, has not been revised since. This could be responsible for the overtriage of trauma patients in Level I Trauma Centers. The principal aim of this study was to evaluate the correlation between each Vittel field triage criterion and trauma patients’ Injury Severity Score.

**Methods:**

Our Level I Trauma Center receives an average of 300 trauma patients per year. Demographic and physiological data, along with the entire trauma patient management process and Vittel field triage criteria, are recorded in a local trauma registry. The Abbreviated Injury Scale (AIS) and Injury Severity Score (ISS) are calculated after a complete assessment of the trauma victim during their in-hospital management. Results were concerned with the presence of an ISS of greater than 15, which defined a major trauma patient; mortality within 30 days; and admission to the intensive care unit. This study is a registry analysis from January 2013 to September 2017.

**Results:**

Of the 1373 patients in the registry, 1151 were included in the analysis with a mean age of 43 years (± 19) and a median ISS of 13 (IQR = 5–22), where 887 (77%) were male. Nine of the 24 Vittel criteria were associated with an ISS > 15. In a multivariate analysis, no criterion related to kinetic elements was significantly correlated with an ISS > 15, mortality within 30 days, or admission to intensive care. Three algorithm categories were predictive of a major trauma patient (ISS > 15): physiological variables, pre-hospital resuscitation, and physical injuries, while kinetic elements were not.

**Conclusions:**

Criteria related to physiological variables, pre-hospital resuscitation, and physical injuries are the most relevant to predicting the severity of a trauma patient’s condition. A revision of the VCA could potentially have beneficial effects on the over and undertriage phenomena, which constitute ongoing medical and financial concerns.

## Background

The goal of pre-hospital triage is to refer an injured patient to the most appropriate center according to their current clinical status. Several studies have proven that referring trauma patients to specialized Trauma Centers (TCs) reduces mortality [1, 2]. The benefits are even greater when patients are in critical condition [3, 4]. Field triage algorithms were initially developed in the United States of America [5], and these provided the basis for the decision of French emergency physicians to create their own triage algorithm for the trauma system in France. In 2002, they developed the Vittel Criteria Algorithm (VCA) for pre-hospital physicians in order to quickly screen possible major trauma patients and refer them to appropriate TCs [6]. However, there was not yet any TC stratification in France at this time, and as time passed, this change resulted in an overtriage of trauma patients in Level I TCs. Pre-hospital overtriage increases costs, patients’ geographical constraints, and overcrowding in referral centers [7].

Consequently, overtriage causes trauma teams to commit valuable resources to patients that do not actually require the care of a Level I TC. This could then lead to an upsurge of patient undertriage in Level I TCs, which increases the morbidity and mortality rates of the most critically ill patients. A few studies have assessed the Vittel criteria, and most of them conclude that there is more overtriage than undertriage [8–10].

**Fig. 1:**
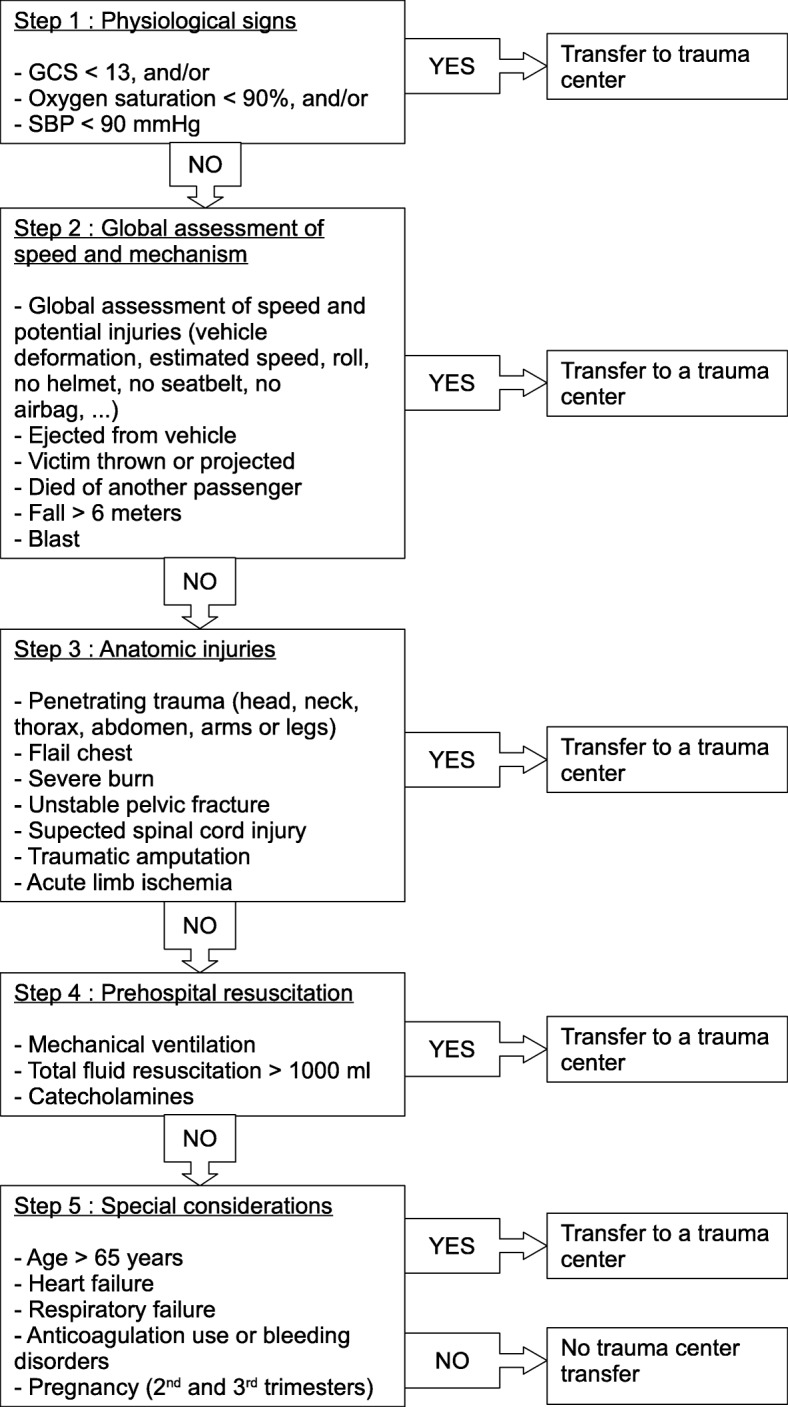
Vittel Criteria Algorithm for French field triage. GCS: Glasgow Coma Scale; SBP: Systolic Blood Pressure

The VCA comprises 24 different criteria divided into five categories: 3 in physiological variables, 6 in kinetic elements, 7 in physical injuries, 3 in pre-hospital resuscitation, and 5 in special considerations (Fig. 1). Pre-hospital resuscitation criteria have been added to the French algorithm due to the rise of pre-hospital emergency care. According to several studies, a good triage algorithm should result in an undertriage of less than 5% with an overtriage of 25–50% [11, 12]. In hospitals, major trauma is usually defined as an Injury Severity Score (ISS) of greater than 15 (gold standard) [13]. This score can only be calculated after a complete evaluation and whole-body CT-scan of the trauma patient in hospital [14]. An appropriate pre-hospital triage protocol would be to direct a major trauma patient (with ISS > 15) to a Level I TC.

Our main objective was to study the correlation between the different pre-hospital field triage criteria and the probability of an ISS of greater than 15. Our secondary objectives were to study their association with mortalities within 30 days and admission to the Intensive Care Unit (ICU).

## Methods

We performed a monocentric registry analysis study in the Sainte Anne Military Hospital of Toulon (South East of France), a Level I TC, which has all the necessary medical and surgical resources for treating any type of traumatic injury. It receives an average of 300 trauma patients per year. A registry of trauma patients in the Sainte Anne hospital in Toulon was started in 2013 to collect data prospectively in accordance with the Utstein-Style guidelines [15]. Raw data were collected prospectively on paper by physicians and were entered into an electronic database. A clinical research assistant regularly verifies the integrity and completeness of the data and collects patient outcomes upon discharge. All patients in the registry from January 2013 to September 2017 were included in the study so long as severe trauma was suspected in the pre-hospital setting according to the Vittel field triage criteria (Fig. 1).

Both demographic and physiological data, along with the entire trauma patient management process (from the scene of the accident to hospital discharge) and Vittel field triage criteria, were recorded in this local trauma registry. The presence of even a single Vittel criterion, which was recorded by the pre-hospital physician in the field, justified the referral of a trauma patient to our TC. There was no severity grading in our criteria. This study was approved by the Sainte Anne Hospital Institutional Review Board (IRB 00011873–2019 – 02).

Our trauma registry consisted of: i) demographic data (age, gender), ii) type of trauma and mechanism of injury; iii) pre-hospital clinical examination results (Glasgow coma scale, heart rate, systolic blood pressure, respiratory rate, oxygen saturation), and iv) pre-hospital resuscitation (mechanical ventilation, catecholamine administration, and fluid loading). When a trauma patient was referred to our major trauma care unit, a whole-body CT-scan was systematically performed. Subsequently, both the Abbreviated Injury Scale (AIS) and Injury Severity Score (ISS) were established after a complete assessment of the trauma victim. The ISS is derived from the AIS which concerns itself with six regions of the human body (head and neck, face, thorax, abdomen, limbs, external surface) [16]. The ISS is then calculated by summing the squares of the three highest AIS ratings. This ISS is then rated from 1 to 75. In common standards, if an injury is rated AIS 6 (fatal), the ISS is arbitrarily set at 75. Our primary outcome was defined by an ISS of greater than 15 (major trauma).

Descriptive statistics included frequencies and percentages for categorical variables, and the mean (SD) or median (IQR) for continuous variables. The association between the 24 field triage criteria and an ISS of greater than 15, a 30-day mortality, and admission to the ICU was analyzed via unifactorial logistic regression. All criteria presenting a *p*-value < 0.20 were systematically included in a multifactorial (adjusted) logistic regression model, where *p* < 0.05 was significant. The results were expressed as an Odds Ratio (OR) with associated confidence intervals (95% CI).

The predictive performance (comprising: Sensitivity (Se), Specificity (Sp), Positive (PPV) and Negative (NPV) Predictive Value, Positive (PLR) and Negative (NLR) Likelihood Ratios, and Youden index) of each of the five VCA categories were calculated.

A Receiver Operating Characteristic (ROC) curve was used in order to assess the VCA predictive performance of having an ISS > 15. The completion of these statistical tests led to the formation of two alternative algorithms. The first was made by eliminating the category with the least predictive performance (i.e. a 4-step algorithm), whereas the second eliminated the two lowest categories (i.e. a 3-step algorithm). Finally, we compared the complete VCA (5 step-algorithm) versus our two alternative 4 and 3-step algorithms in accordance with the method of Delong et al. [17].

The statistical software SPSSv20 and MedCalc V14.8.1 were used for data acquisition and analysis. The significance was set at *p* < 0.05.

## Results

**Fig. 2:**
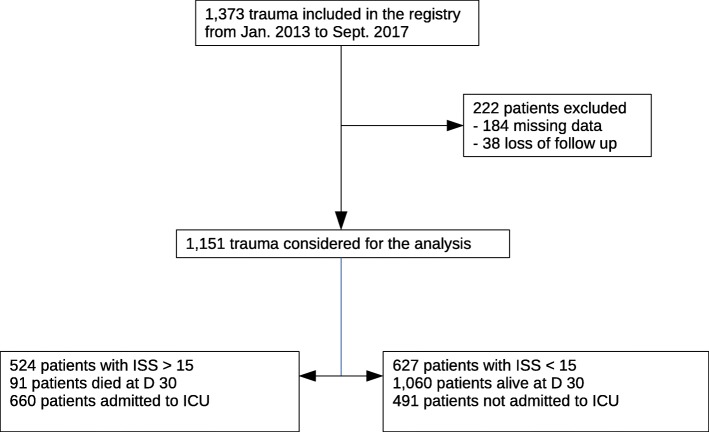
Study population flowchart. ISS: Injury Severity Score, D: Day, ICU: Intensive Care Unit

Between January 2013 and September 2017, 1373 trauma patients were added to the TC’s registry, of which 222 (16%) were excluded from the study due to either missing data (184 patients) or loss to follow up (38 patients) (Fig. 2). This left 1151 (84%) includable patients that were analyzed in the study. On average, each patient met 2 to 3 field triage criteria. Twenty-seven (2%) patients did not meet any field triage criteria. The main characteristics of the study population are listed in Table 1.

**Table 1:** Study population characteristics (*n* = 1151 patients)

Characteristic	Value
Sex: Male, *n* (%)	887 (77)
Age: mean (SD)	43 (±19)
Mechanism of injury	
Blunt, *n* (%)	1063 (92)
Penetrating, *n* (%)	88 (8)
Type of trauma	
Car crash, *n* (%)	263 (23)
Motorcycle, *n* (%)	457 (40)
Pedestrian, *n* (%)	74 (6)
Fall, *n* (%)	228 (20)
Gunshot, *n* (%)	48 (4)
Stab wound, *n* (%)	34 (3)
Other, *n* (%)	47 (4)
Pre-hospital physiological variables: median (IQR)	
Glasgow coma scale	15 (13–15)
Heart rate, beats/min	86 (75–100)
Systolic arterial blood pressure, mm Hg	120 (108–137)
Peripheral oxygen saturation, %	99 (97–100)
Pre-hospital resuscitation	
Mechanical Ventilation, *n* (%)	197 (17)
Catecholamine administration, *n* (%)	106 (9)
Total fluid loading, mL: median (IQR)	250 (0–500)
MGAP score: median (IQR)	27 (22–28)
MGAP category, *n* (%)	
3–17 ^a^	133 (12)
18–22 ^b^	201 (18)
23–29 ^c^	817 (71)
ISS: median (IQR)	13 (5–22)
ISS ≥ 15, *n* (%)	524 (46)
Field triage criterion categories, *n* (%)	
Physiological variables	420 (15)
Kinetic elements	1328 (48)
Physical injuries	383 (14)
Pre-hospital resuscitation	373 (13.5)
Special considerations	260 (9.5)
ICU admission during the hospital stay, *n* (%)	660 (57)
In-hospital mortality within 30 days, *n* (%)	91 (8)

MGAP score: Mechanism, Glasgow coma scale, Age, systolic blood Pressure

^a^High risk of mortality; ^b^intermediate risk of mortality; ^c^low risk of mortality

*ISS* Injury Severity Score, *ICU* Intensive Care Unit

### Primary results

**Fig. 3:**
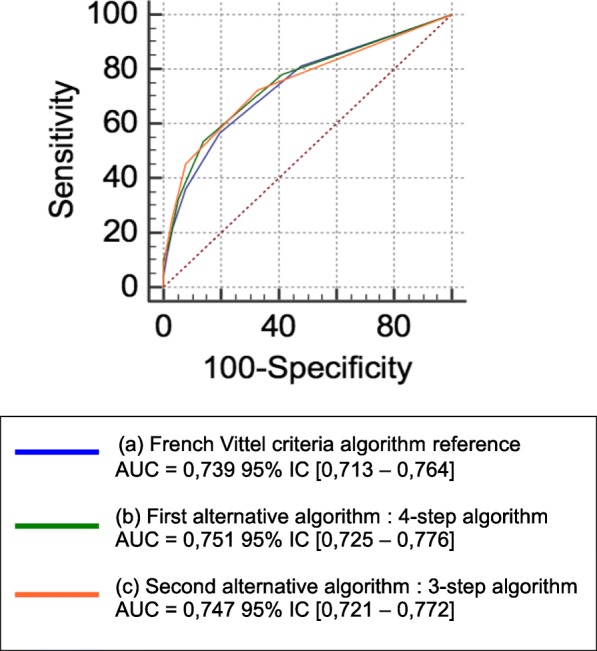
ROC curves illustrating the predictive performance of each of the 3 algorithms concerning an ISS > 15. (**a**) French Vittel Criteria Algorithm (VCA) reference (**b**) First alternative algorithm, a 4-step algorithm: VCA without kinetic elements (2nd category) (**c**) Second alternative algorithm, a 3-step algorithm: VCA without kinetic elements (2nd category) or special considerations (5th category)

In our Level I TC, 524 patients (46%) presented an ISS of greater than 15. The correlation between the field triage criteria and an ISS > 15 is reported in Tables 2 and 3 (univariate and multivariate analysis, respectively). Nine field triage criteria were significantly associated with an ISS of greater than 15 in multivariate analysis (*p* < 0.05). Table 4 includes the predictive performance of each category of the VCA regarding an ISS of greater than 15. Physiological variables (1st category) and pre-hospital resuscitation (4th category) had greater predictive performance concerning an ISS > 15. However, kinetic elements (2nd category) had the poorest performance. ROC curve comparisons did not find any significant differences between the VCA and our 3 and 4-step alternative algorithms (Fig. 3 and Table 5).

**Table 2:** Association between each field triage criterion and risk of an ISS > 15, mortality, or admission to intensive care, respectively, in univariate analysis

Field triage criteria	*N* = 2764^a^	Evaluation criterion, OR (95% CI)				
		ISS > 15	30-day mortality	Admission to ICU		
GCS score < 13	252	7.9 (5.6–11.2)	17.2 (10.2–29)	8.5 (5.6–12.9)		
SBP < 90 mmHg	105	4.1 (2.6–6.5)	3 (1.7–5.2)	4.3 (2.5–7.3)		
Oxygen saturation < 90%	63	4.5 (2.5–8.3)	9.4 (5.3–16.5)	3.3 (1.8–6.3)		
Ejection from vehicle	112	0.9 (0.6–1.3)	0.9 (0.4–1.9)	1.4 (0.9–2.1)		
Death of another passenger	31	1.5 (0.7–3)	0.8 (0.2–3.4)	3.2 (1.3–7.8)		
Fall > 6 m	228	1.3 (1–1.8)	1.5 (0.9–2.5)	1.1 (0.8–1.4)		
Victim thrown or run over	253	1.1 (0.8–1.4)	1.1 (0.7–1.9)	1.4 (1.02–1.8)		
Global assessment (speed or vehicle deformation)	701	0.9 (0.7–1.1)	0.6 (0.4–0.8)	1 (0.8–1.2)		
Blast	3	0.6 (0.1–6.6)	NC	0.4 (0.03–4.1)		
Penetrating trauma	88	1.1 (0.7–1.6)	3.2 (1.8–5.7)	1 (0.7–1.6)		
Flail chest	48	9 (3.8–21.4)	2.1 (0.9–4.8)	3.4 (1.6–7)		
Severe burn	4	1.2 (0.2–8.5)	3.9 (0.4–38)	NC		
Pelvis fracture	72	1.6 (1.01–2.6)	0.9 (0.3–2.2)	2 (1.2–3.4)		
Spinal cord injury	137	2.5 (1.7–3.6)	3 (1.8–5)	2.7 (1.8–4.1)		
Amputation	17	2.9 (1.02–8.3)	2.6 (0.7–9)	3.5 (1.01–12.3)		
Acute limb ischemia	17	1.7 (0.7–4.6)	0.7 (0.1–5.5)	1.4 (0.5–3.7)		
Mechanical ventilation	197	8.9 (6–13.4)	13.8 (8.6–22.1)	19.1 (10–36.5)		
Total fluid resuscitation > 1000 mL	70	8 (4–15.8)	2.9 (1.5–5.6)	5.5 (2.7–11.1)		
Catecholamine administration	106	10.2 (5.7–18.5)	10.4 (6.4–16.9)	14.5 (6.3–33.4)		
Age > 65 years	192	2 (1.5–2.8)	4.7 (3–7.3)	1.5 (1.1–2.1)		
Heart failure	29	1.3 (0.6–2.7)	2.5 (0.9–6.7)	1.4 (0.7–3.1)		
Respiratory failure	4	1.2 (0.2–8.5)	NC	0.7 (0.1–5.3)		
Pregnancy	4	1.2 (0.2–8.5)	NC	0.3 (0.03–2.4)		
Anticoagulant use or bleeding disorders	31	1.3 (0.6–2.6)	3.6 (1.5–8.6)	1.4 (0.7–2.9)		

*GCS* Glasgow Coma Scale, *SBP* Systolic Blood Pressure, *ISS* Injury Severity Score, *OR* Odds Ratio, *NC* Not Calculable

^a^*N* = total number of triage criteria recorded within the study population (*n* = 1151)

**Table 3:** Association between each field triage criterion and risk of an ISS > 15, mortality, or admission to intensive care, respectively, in multivariate analysis

Field triage criteria	Evaluation criterion, OR (95% CI)				
	ISS > 15	30-day mortality	Admission to ICU		
GCS score < 13	4.2 (2.7–6.6)	7.6 (3.6–16.2)	3.4 (2.1–5.5)		
SBP < 90 mmHg	2.3 (1.3–3.9)	NS	2.3 (1.3–4.3)		
Oxygen saturation < 90%	NS	5.6 (2.8–11.6)	NS		
Flail chest	8 (3.2–19.9)	NS	2.8 (1.3–6.1)		
Pelvis fracture	NS	NS	2.2 (1.3–3.9)		
Amputation	3.4 (1.1–10.4)	NS	NS		
Spinal cord injury	1.9 (1.2–2.9)	NS	2.1 (1.3–3.3)		
Penetrating trauma	NS	3.2 (1.5–7.1)	NS		
Mechanical ventilation	2.2 (1.3–3.8)	4.1 (1.8–9)	5.9 (2.8–12.4)		
Total fluid loading > 1000 mL	4 (1.9–8.5)	NS	NS		
Catecholamine administration	2 (1.01–4.1)	2.3 (1.2–4.4)	2.8 (1.1–7.1)		
Age > 65 years	2 (1.4–2.8)	7.6 (4–14.4)	1.5 (1.02–2.1)		
Anticoagulant use or bleeding disorders	NS	3.8 (1.1–10.9)	NS		

*GCS* Glasgow Coma Scale, *SBP* Systolic Blood Pressure, *ISS* Injury Severity Score, *OR* Odds Ratio, *NS* Not Significant

**Table 4:** Performance of each category of the Vittel Criteria Algorithm (VCA) in predicting the risk of an ISS > 15, mortality within 30 days, or admission to Intensive Care Unit

VCA Category	*N* = 2764^a^	Predictive performance for an ISS > 15						
		Se %	Sp %	PPV %	NPV %	PLR	NLR	Youden index
Step 1: Physiological variables	420	50	87	77	68	3,9	0.57	0.37
Step 2: Kinetic elements	1328	86	13	45	52	0.99	1.09	−0.01
Step 3: Physical injuries	383	41	77	60	61	1.77	0.77	0.18
Step 4: Pre-hospital resuscitation	373	41	94	84	65	6.19	0.68	0.34
Step 5: Special considerations	260	23	86	58	57	1.27	0.85	0.09
VCA Category	*N* = 2764^a^	Predictive performance for 30-day mortality						
		Se %	Sp %	VPP %	VPN %	PLR	NLR	Youden index
Step 1: Physiological variables	420	89	75	24	99	3.57	0.15	0.64
Step 2: Kinetic elements	1328	77	13	7	86	0.88	1.85	−0.11
Step 3: Physical injuries	383	54	71	14	95	1.87	0.65	0.25
Step 4: Pre-hospital resuscitation	373	73	82	26	97	4.11	0.33	0.55
Step 5: Special considerations	260	45	84	20	95	2.8	0.7	0.29
VCA Category	*N* = 2764^a^	Predictive performance for admission to ICU						
		Se %	Sp %	VPP %	VPN %	PLR	NLR	Youden index
Step 1: Physiological variables	420	45	90	86	55	4.49	0.61	0.35
Step 2: Kinetic elements	1328	86	12	57	39	0.98	1.19	−0.02
Step 3: Physical injuries	383	38	78	70	48	1.75	0.79	0.16
Step 4: Pre-hospital resuscitation	373	36	97	93	53	10.3	0.7	0.32
Step 5: Special considerations	260	20	85	64	44	1.3	0.9	0.05

*Se* Sensibility, *Sp* Specificity, *PPV* Positive Predictive Value, *NPV* Negative Predictive Value, *PLR* Positive Likelihood Ratio, *NLR* Negative Likelihood Ratio

^a^*N* = total number of triage criteria within the study population (*n* = 1151)

**Table 5:** Comparison between the predictive performance of the Vittel Criteria Algorithm (VCA) for an ISS > 15 and of our two alternative algorithms

Pre-hospital field triage algorithms	AUC difference	95% CI	*p* value
VCA vs. 4-step algorithm	0.012	−0.004 – 0.028	0.15
VCA vs. 3-step algorithm	0.008	−0.012 – 0.029	0.43
4-step algorithm vs. 3-step algorithm	0.004	−0.009 – 0.016	0.59

*AUC* Area Under Curve

*VCA* Vittel Criteria Algorithm

### Secondary results

Six hundred and sixty patients (57%) were admitted to the ICU. The 30-day mortality rate was 8% of the entire study population, and 16% of trauma patients with an ISS > 15. In a multivariate analysis, 7 and 8 Vittel field triage criteria were significantly associated with the 30-day mortality rate and ICU admissions, respectively (Table 3). Similarly, physiological variables and pre-hospital resuscitation had the best performances in predicting the 30-day mortality rate and the necessity for admission to an ICU. Kinetic elements had the poorest predictive performance (Table 4). The analysis of the VCA ROC curves revealed an AUROC of 0.87 (0.85–0.89) for predicting a death within 30 days and an AUROC of 0.74 (0.71–0.76) for predicting an admission to the ICU.

## Discussion

Of the 24 Vittel field triage criteria, 9 were associated with the probability of having an ISS of greater than 15. Two categories of the VCA were strongly predictive of a major trauma patient (ISS > 15): physiological variables and pre-hospital resuscitation. Physical injuries also constituted a good indicator for predicting an ISS of greater than 15, whereas kinetic elements were not. The comparison of our two alternative algorithms’ ROC curves with the VCA did not reveal any differences in predicting an ISS of greater than 15. Regarding our secondary results, 7 and 8 criteria were associated with a risk of 30-day mortality and ICU admission, respectively. The most relevant categories for predicting the risk of 30-day mortality and ICU admission were also physiological variables, pre-hospital resuscitation, and physical injuries, while kinetic elements did not appear to be significant.

In general, major trauma patients (ISS > 15) usually meet several field triage criteria. In our study, major trauma patients mainly presented kinetic elements, in combination with other algorithmic categories (physiological variables, pre-hospital resuscitation, or physical injuries). However, the kinetic elements category of field triage may not be the most relevant for identifying patients who will require the resources of a Level I TC [18]. What’s more, we demonstrated that kinetic elements were not significant indicators of patients with an ISS of greater than 15. In our study, the elimination of the criteria related to kinetic elements (2nd category; 4-step algorithm) and/or special considerations (5th category; 3-step algorithm) did not improve the performance of the algorithm in predicting an ISS > 15 in trauma patients (no significant differences between AUROCs). Our results highlighted the limitations of some of the components of the current triage algorithm in defining severe trauma. Indeed, it could be interesting to further assess the effect of score adjustments on the pre-hospital misclassification of trauma patients.

In their study, Hamada et al. also found a weak association between the ISS and kinetic elements criteria (PLR = 0.9, NLR = 1.2) [9], whose poor predictive performance was also underlined by the American College of Surgeons Committee On Trauma (ACSCOT) in 2006. In fact, the revision of American field triage algorithms led to the modification of the kinetic elements’ category (i.e. the removal of several criteria such as the initial speed, vehicle deformity, and the extrication duration). Lerner et al. (2011) demonstrated that a revision of their current algorithm significantly reduced triage errors by 11% (34% in 1999 versus 23% in 2006). As was to be expected, their reduction in overtriage had the subsequent effect of a small increase in the number undertriage patients [19].

The relevance of field triage criteria is inextricably linked to the nature and organization of the associated trauma system, and the field triage criteria related to physiological variables and pre-hospital resuscitation change over time. Therefore, it would also be interesting to evaluate the relevance of the triage criteria of these two categories as a function of arrival times at the trauma center (more or less than 30 min of emergency medical transportation). Brown et al. (2011) reported that current physiological variables and physical injuries criteria described within the American national trauma triage protocol are both very specific and predictive of the need of referral to a TC [20]. In a trauma system where TCs are stratified, referring trauma patients exclusively suffering from physiological variables, physical injuries, and/or pre-hospital resuscitation to Level I TCs and redirecting those presenting only kinetic elements-related criteria toward lower levels (II or III) may reduce triage errors [21].

In hospitals, the Injury Severity Score is the most frequently used indicator of trauma severity [22]. This score is mostly based on physical injuries and does not assess the need for specific technical resources. Consequently, the use of composite criteria such as the need for massive transfusions, interventional radiology, neurosurgery, or specialized damage control management (surgical and resuscitative) would be more suitable for referring a trauma patient to a Level I TC [9, 23–25]. In our study, the number of trauma patients admitted to the ICU was greater than the number of patients with an ISS > 15. Several other pre-hospital triage scores have been developed to assess the severity of trauma patients (T-RTS, MGAP score, NTS) [26–28], and while these scores are based on physiological variables and assess the mortality of trauma patients, they do not appear to have a good correlation with the ISS (AUROCs for pre-admission RTS and MGAP were 0.64 and 0.67, respectively) [29].

An age of greater than 65 years old was identified as a risk factor for having an ISS > 15 [OR = 2 (1.4–2.8)], death within 30 days [OR = 7.6 (4–14.4)], and admission to intensive care units [OR = 1.5 (1.02–2.1)]. These results are in agreement with the literature [30–32]. Particular consideration should be given to these elderly patients, who may not present physiological variables at the time of initial management but whose mortality rate is higher than that of younger patients [33]. Early management by a specialized trauma team could reduce their in-hospital mortality rate [34]. Field triage algorithms constitute a valuable aide for pre-hospital teams but should not be a substitute for clinical common sense. It is important to be aware of all the technical resources provided by TC platforms in order to ensure optimal management and to limit undertriage as much as possible [35]. Further studies are required in order to compile exhaustive and optimal pre-hospital triage criteria or algorithms that can aide physicians in their decision-making for appropriate referrals of a trauma patient to their right place at the right time [36].

This study has several limitations. Firstly, this is a monocentric study in a Level I TC. Our study focused on patients who met pre-hospital VCA criteria and were referred to our Level I TC. We did not consider patients who met pre-hospital VCA criteria but, for logistical reasons or initial triage errors, were referred to lower level TCs (II or III), nor those who did not meet at least one VCA criterion during the pre-hospital examination and were directed towards Level II or III TCs yet had an ISS > 15 after a whole-body CT-scan. Thus, our study does not allow for the assessment of undertriage with VCA use. Secondly, our study did not allow us to determine whether the decision of the emergency response services or initial physician was only made based on VCA criteria, nor whether the algorithm was used correctly or not. Thirdly, a large number of patients were excluded (*n* = 222) due to either a lack of essential data in the registry concerning pre-admission and/or hospital stay (*n* = 184), or loss to follow-up due to transfer to another hospital (*n* = 38).

## Conclusion

Numerous criteria are used by field triage algorithms to evaluate trauma patients. Each criterion of the VCA has a more or less powerful association with the risk of having an ISS of greater than 15. Criteria related to physiological variables, pre-hospital resuscitation, and physical injuries are the most relevant to predicting the severity of trauma. Criteria related to kinetic elements (in particular those related to vehicle deformation, estimated speed, and ejection from said vehicle) were not significant predictors of trauma severity. A revision of the French triage algorithm, due to its potential effects on the over and the undertriage phenomena, could hold benefits of individual and collective interest, as the misclassification of patients introduces both geographical constraints and medical and financial concerns.

## Data Availability

The data that support the findings of this study are available upon request from the corresponding author, JB, or EM. The data contain information that could compromise research participant privacy and, therefore, are not publicly available.

## Abbreviations

AIS: Abbreviated Injury Score
AUC: Area Under Curve
AUROC: Area Under the Receiver Operating Characteristics
CI: Confidence Intervals
ICU: Intensive Care Unit
IRB: Institutional Review Board
ISS: Injury Severity Score
MGAP score: Mechanism, Glasgow coma scale, Age, arterial Pressure
NTS: New Trauma Score
OR: Odds Ratio
ROC: Receiver Operating Characteristic
RTS: Revised Trauma Score
TC: Trauma Center
T-RTS: Triage-Revised Trauma Score
